# *Candida* Infection Associated with Salivary Gland—A Narrative Review

**DOI:** 10.3390/jcm10010097

**Published:** 2020-12-30

**Authors:** Soo-Min Ok, Donald Ho, Tyler Lynd, Yong-Woo Ahn, Hye-Min Ju, Sung-Hee Jeong, Kyounga Cheon

**Affiliations:** 1 Department of Oral Medicine, Dental and Life Science Institute, Pusan National University, Yangsan 50612, Korea; oksoomin@pusan.ac.kr (S.-m.O.); ahnyongw@pusan.ac.kr (Y.-W.A.); hyungtaejoa@naver.com (H.-M.J.); drcookie@pusan.ac.kr (S.-H.J.); 2 Dental Research Institute, Pusan National University Dental Hospital, Yangsan 50612, Korea; 3 Department of Pediatric Dentistry, University of Alabama at Birmingham, Birmingham, AL 35294, USA; donaldho@uab.edu (D.H.); tlynd@uab.edu (T.L.)

**Keywords:** oral candidiasis, salivary gland, saliva, treatment

## Abstract

*Candida* species are common global opportunistic pathogens that could repeatedly and chronically cause oral mucosa infection and create an inflammatory environment, leading to organ dysfunction. Oral *Candida* infections may cause temporary or permanent damage to salivary glands, resulting in the destruction of acinar cells and the formation of scar tissue. Restricted function of the salivary glands leads to discomfort and diseases of the oral mucosa, such as dry mouth and associated infection. This narrative review attempts to summarize the anatomy and function of salivary glands, the associations between *Candida* and saliva, the effects of *Candida* infection on salivary glands, and the treatment strategies. Overall, clinicians should proactively manage *Candida* infections by educating patients on oral hygiene management for vulnerable populations, conducting frequent checks for a timely diagnosis, and providing an effective treatment plan.

## 1. Introduction

Salivary glands, an essential component to maintaining oral health, are susceptible to a variety of pathologies, including candidiasis. The salivary glands are commonly classified as either major or minor salivary glands based on their sizes, distributions, and functional characteristics [1]. The major salivary glands consist of the parotid, submandibular, and sublingual glands [2], which produce and secrete saliva, moisturize intraoral mucosa and teeth, maintain oral hygiene, and facilitate taste, swallowing, speech, and mastication [3]. The minor salivary glands are distributed throughout oral mucosa surfaces, producing mucous saliva with organic substances, even at night, and protect oral mucosa from injury [4,5,6]. Notably, salivary glands produce high concentrations of the secretory immunoglobulin (Ig) A, which prevents other Igs from being broken down by proteolytic enzymes from microbes [7,8]. These critical functions of saliva are repressed when the salivary glands are damaged by *Candida* infections.

*Candida* is a genus of yeast and major human fungal pathogens [9]. *Candida* species are opportunistic pathogens that could repeatedly and chronically cause oral mucosa infections [10,11]. The most prevalent species found in oral *Candida* infection is *Candida albicans*, due to its cell adherence properties and great pathogenic potential [12]. *C. albicans* is isolated from more than 80% of oral *Candida* lesions [13]. Other clinically relevant species include *Candida glabrata*, *Candida *tropicalis*, Candida *parapsilosis*, Candida kefyr, Candida *dubliniensis*, Candida *lusitaniae*, Candida *krusei,** and Candida guilliermondii [14]. It has been reported that 30–45% of healthy adults carry oral *Candida* organisms, and 25–80% of adults develop oral candidiasis under the condition of using antibiotics, steroids, or immunosuppressants; impaired salivary gland function; improperly fitted dentures; poor oral hygiene; and a high carbohydrate diet. Additionally, 49–54% of healthy infants carry oral *Candida* organisms, and 5–7% of infants develop oral candidiasis [15,16]. In general, the most commonly affected populations are middle-aged to elderly people. Prevalence rates as high as 70% have been reported in nursing-home residents [17]. Denture-associated oral candidiasis is frequent and occurs globally. Additionally, females are affected slightly more frequently than males [17]. Oral candidiasis also occurs in immunocompromised patients, with an estimated prevalence of 9–31% of acquired immunodeficiency syndrome (AIDS) patients and 20% of cancer patients [18]. 

Host inflammatory reaction to *Candida* infection may negatively affect salivary gland tissue and function. During *Candida* infection, epithelial leukocyte penetration and subepithelial inflammation are observed in histological examinations [19]. The inflammatory mediators, such as chemokines and cytokines (TNF-α, IL-6, and IL1β), are secreted from oral epithelial cells and phagocytic cells, including neutrophils, macrophages, and dendritic cells [19]. The inflammatory reaction could damage salivary glands in the form of sialectasis, ductal ectasia, and progressive acinar destruction. The sublingual and minor salivary glands are located in the superficial layer of the oral mucosa and may be more vulnerable to inflammatory-mediated damage. 

Based on clinical observation and pathological evidence from the literature, this review article discusses the anatomy and function of the salivary gland, the association between *Candida* and saliva, the effects of oral *Candida* infection on salivary glands, and treatment strategies to combat *Candida* infection. 

## 2. Anatomy and Function of the Salivary Glands

**Figure 1:**
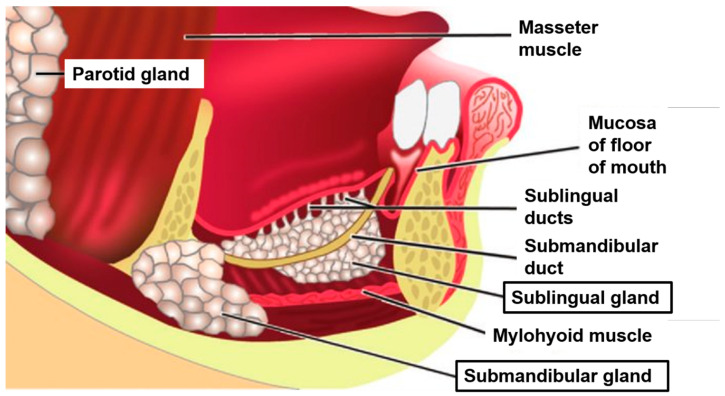
Drawing shows the location of the major salivary glands and its ducts. Note: Sublingual gland is located below the oral epithelium of the floor of the mouth. Adapted with permission of Radiological Society of North America, from “Imaging the Floor of the Mouth and the Sublingual Space”, 31, 5, 2011 [23]; permission conveyed through Copyright Clearance Center, Inc.

To understand the implications of *Candida* infection and how it affects salivary glands, the anatomy and function of normal salivary gland are described. As shown in Figure 1, there are three major salivary glands: parotid, submandibular, and sublingual glands in the oral cavity. The paired parotid glands are the largest of the major salivary gland; they are encapsulated and located lateral to the ramus of the mandible [20]. The parotid gland consists mainly of serous acini, secreting α-amylase-rich saliva. Saliva α-amylase is known to play a secondary role in preventing bacterial attachment to the oral surface and removing bacteria from the oral cavity [21]. The paired submandibular glands are the second largest salivary gland, located in the submandibular triangle, consisting of anterior and posterior digastric muscles, and lower border of the mandible, making up the posterior part of the floor of the mouth, above the mylohyoid muscle. The submandibular glands are composed of mixed acini populations with mucous and serous function [20]. The sublingual glands are the smallest major salivary gland and are located right under the mucous membrane at the floor of the mouth [22]. Unlike the parotid and submandibular glands, the sublingual glands are not encapsulated and spread throughout the sublingual space. The sublingual space is just below the floor of mouth and above the mylohyoid muscles. The sublingual glands secrete mucous saliva, a viscous solution rich in mucins. While the parotid and submandibular glands have long branched ducts containing all the ductal segments (excretory, intercalated, and striated), the sublingual glands lack striated ducts. The three main salivary glands account for more than 90% of secreted saliva by volume.

**Figure 2:**
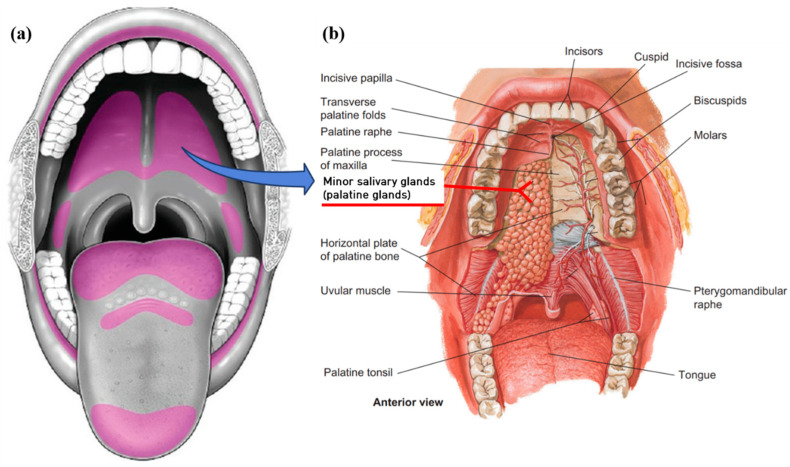
(**a**) Minor salivary gland distribution in the oral cavity (shown as purple shade). Adapted with permission of Wolters Kluwer Medknow Publications from Review of the Major and Minor Salivary Glands, Part 1: Anatomy, Infectious, and Inflammatory Processes, 8, 1, 2018 [31]. (**b**) Minor salivary gland are located just below the oral epithelium (Adapted with permission of Elsevier Science and Technology Books from *Comparative Anatomy and Histology: A Mouse, Rat, and Human Atlas*, 2017 [32]). (**a**,**b**) Permission conveyed through Copyright Clearance Center, Inc.

Minor salivary glands are located in the submucosa, where they are surrounded by connective tissue, or embedded between muscle fibers (Figure 2). Between 600 and 1000 minor salivary glands are scattered throughout the oral mucosa except for the gingiva, the anterior dorsal aspect of the tongue, the midline and anterior part of the hard palate. Minor salivary glands consist of small secretory cell clusters with short excretory ducts that convey saliva products to the mucosal surface [4]. Minor salivary glands have a diameter of 1–5 mm and no actual capsule like the sublingual gland. Most of the minor salivary glands secrete mucus saliva; however, Von Ebner glands secrete serous or mixed saliva. The Von Ebner glands are adjacent to the foliate and circumvallate papillae in the dorsum and posterior tongue [4]. Although minor salivary glands produce about 10% of the total saliva volume [24], the minor salivary glands are widely distributed throughout the oral submucosa and secrete an abundance of salivary mucins, which acts as a lubricant. Mucin is important component of saliva, to avoid the subjective sensation of oral dryness [6]. By secretion of salivary mucins from the minor salivary glands, the formation of a lubricating film on the oral surfaces contributes to mucosal wetting and protection [5]. Meanwhile, researchers demonstrated that the flow rate of the minor salivary glands is a critical factor for dry-mouth assessment [25,26]. Since subjective feelings from dry mouth are associated with decreased flow rates of the minor salivary gland, the minor salivary gland flow rate could be used a xerostomia-assessment tool [25,26]. Furthermore, minor salivary glands produce saliva during sleep. It appears that a reduced minor salivary gland flow rate could account for dry mouth at night [27]. By secretion of salivary mucins from the minor salivary glands, the formation of a lubricating film on the oral surfaces contributes to mucosal wetting and protection [5]. Minor salivary glands also secrete high concentrations of antibacterial components, such as IgA, to protect the oral mucosa [28]. More than a third of secretory IgA is secreted from the minor salivary glands in whole saliva [29]. Secretory IgA enhances the antibacterial activity of mucin, lactoferrin, peroxidase, and aglutinin [30]. 

Whole saliva secreted by both major and minor salivary glands is an essential fluid for oral maintenance and function. Saliva initiates the digestive process with digestive enzymes, while simultaneously lubricating the solid diet, to assist with passage through the esophagus. Saliva plays an important role in pronunciation and taste by moisturizing the tongue and other tissues in the mouth. Saliva maintains the acid–base balance of the oral cavity, to protect teeth and soft tissues from prolonged acid exposure due to diet and gastroesophageal reflux [33,34]. Additionally, whole saliva contains several signaling molecules essential for the regeneration of oral and esophageal mucosa, including epidermal growth factor, fibroblast growth factor, nerve growth factor, and transforming growth factor alpha. Furthermore, lactoferrin, saliva’s Ig, and lysozyme inhibit the progression of oral bacterial or fungal infections [33]. Due to their location directly below the mucosa, the sublingual gland and the minor salivary glands are susceptible to mucosal infection. Therefore, saliva from the major and minor salivary glands prevents oral mucosal diseases, maintains oral hygiene, and lubricates the oral cavity.

## 3. Pathology of Salivary Glands

### 3.1. Infectious Diseases Involving Virus, Bacteria, and Fungi

#### 3.1.1. Viral and Bacterial Infectious Diseases

Salivary gland tissue may become infected with numerous viruses and bacteria, thus increasing the susceptibility of candidiasis. Epidemic parotitis is caused by the *Paramyxovirus* (mumps is the most common infection) and develops parotid edema with systemic symptoms, including fever, anorexia, malaise, and headache [35]. HIV infects the parotid gland and can lead to the formation of benign lymphoepithelial cystic lesions. *Coxsackie* virus and Hepatitis C virus are RNA-bound viruses that can infect the salivary glands and damage host tissues, causing dry mouth [35]. *Cytomegalovirus* (CMV) is a widespread virus with symptoms ranging from asymptomatic to severe-end organ dysfunction. Human CMV infection, usually affecting salivary glands, could be asymptomatic in healthy people, or it could be a life-threatening viral infection to immunocompromised individuals [36]. Recent evidence suggests that salivary glands are a potential target of severe acute respiratory syndrome coronavirus 2 (SARS-CoV-2) infection [37]. The study explains that SARS-CoV-2 infection was found in the salivary glands by identifying the presence of angiotensin I–converting enzyme 2 and cellular protease transmembrane serine protease 2 in the salivary glands [38]. Furthermore, many researchers reported sialadenitis by SARS-CoV-2 infection and the importance of saliva as a diagnostic tool [39,40,41]. In minor salivary glands, various viral infections also have been reported, such as Epstein–Barr virus, HIV, and human T-lymphotropic virus [42,43,44]. 

Bacterial infections are comparatively rare to viral infections. These infections are caused by either a ductal obstruction or a retrograde spread of infection up the duct secondary to decreased salivary flow. Salivary gland bacterial infections develop in patients who have existing conditions, including postoperative recovery, diabetes or immunodeficiency. Radiation therapy or antidepressant medication could reduce salivary flow and consequently induce staphylococcal and streptococcal strains associated with the biofilm on oral mucosa to infect the salivary gland [20]. Untreated bacterial infections could spread beyond the glandular borders and extend between the neck muscles, leading to serious complications, such as sepsis. In attempt to combat bacterial infection, immune cells infiltrate into the salivary gland and may destroy the secretory system resulting in dry mouth, local pain, and edema [45]. Mycoplasma infection affecting the minor salivary glands has been reported to damage the ductal epithelium, disrupt acinar structures, and cause mucus outflow into connective tissue [46]. Salivary gland dysfunction and destruction caused by various viral and bacterial infection can create a vulnerable condition that causes *Candida* infection in the oral mucosa and even in the salivary glands.

#### 3.1.2. Infectious Diseases with *Candida*

A study reported that *Candida* infection of the parotid gland among healthy adults was caused by deep wounds from dentures [47]. Importantly, dentures were found to disrupt the epithelial barrier and induce *Candida* infection, infiltrating below the mucous membrane. Although fungal infection is reported less frequently, *C. albicans*, *Histoplasma capsulatum*, and *Cryptococcus neoformans* are the most common causes of sialadenitis, salivary gland inflammation [48,49,50,51,52,53,54]. Signs and symptoms of sialadenitis by *C. albicans* are low grade fever (37.4–37.8 °C), painful inflammatory-mediated swelling, and salivary gland duct discharge [10]. Histopathologically, the pus from the parotid gland demonstrated budding yeast cells. The yeast-like cells were between 2 and 4 μm in length within the intra- and extra-cellular space of macrophages [10]. Mostly, salivary gland infection with *Candida* occurs in individuals with impaired immunity, including persons impacted by HIV/AIDS [55]. Additionally, diabetic patients were reported to have increased susceptibility to *Candida* associated parotitis [10,48]. 

### 3.2. Non-Infectious Inflammatory Disease

Sjögren’s syndrome is an autoimmune disorder that causes chronic inflammation and fibrosis of the salivary glands. The primary presenting symptoms of Sjögren’s syndrome are dry eyes and mouth. A study reported that Sjögren’s syndrome is associated with the IgG4 spectrum of disease [46]. As the disease progresses and becomes chronic, salivary glands show atrophy, parenchymal calcification, fat replacement, cystic destruction, or multiple lymphocyte cysts [47]. Chronic Sjögren’s sialadenitis demonstrates ductal stenosis and swelling using sialography [56]. Chronic sclerosis sialadenitis, also known as Küttner’s tumor, indicates chronic enlargement of the salivary glands caused by immune-mediated infiltration of lymphoplasmacytic cells [57]. Remarkably, recent works in the literature have shown strong associations with IgG4-related plasma cell infiltration in over 90% of patients with chronic sclerosing sialadenitis [58]. Mikulicz disease’s cause is unknown, but it is believed to be autoimmune. The disease resembles Sjögren’s syndrome except the fact that salivary and lacrimal secretion is less than that of Sjögren’s syndrome [20]. Sarcoidosis is an autoimmune disease of unknown etiology and can affect multiple systems of the body. Large granulomas can develop in the salivary glands. The granulomas appear as masses with several non-cavity characteristics [45]. The loss of salivary function associated with these inflammatory diseases may cause consequent *Candida* infection. 

### 3.3. Secondary Candida Infection with Salivary Gland Tumors

Salivary gland tumors are often manifested as painless, slow growing, and benign. However, 20% of salivary gland tumors are diagnosed as malignant tumor. Salivary gland tumors are indicated to be benign in 85% of tumors affecting the parotid, 60% of tumors affecting the submandibular, 50% of tumors affecting the minor glands, and only 10% of tumors affecting the sublingual glands [59]. Several studies have reported the association between Epstein–Barr virus infection and salivary gland neoplasm. Epstein–Barr virus may be a major factor in its etiology or pathogenesis [55,60,61,62]. HIV infection was also found to increase the risk of salivary gland cancers. Salivary gland neoplasms, such as adenoid cystic carcinoma, Kaposi sarcoma, and lymphoma, are reported in HIV infection [63]. 

*Candida* infection associated with salivary gland neoplasm has been rarely reported. However, *Candida* infection in the development of squamous cell carcinoma has been suspected for years. Common sites for oral squamous cell carcinoma to develop are on the tongue, lips, and floor of the mouth, where minor salivary glands and sublingual and submandibular glands are distributed. *Candida* species are more prevalent in potentially malignant oral mucosa diseases and cancerous oral mucosa lesions than in mucosa with the non-cancerous diseases [64,65]. With *Candida* infection, the rate of malignant transformation in leukoplakia is higher than in leukoplakia without candidiasis. The susceptibility to *Candida* infection often relies on an imbalance between *Candida* virulence factors and the host’s defensive immune system [66]. In order for *C. albicans* to penetrate into mucosa, cell surface proteins, called adhesins, must recognize host molecules and adhere to the host cell. After they adhere to the cell surface, the cellular phenotype converts from yeast form to hyphae form by two mechanisms. The first mechanism is the secretion of a protease, a degrading enzyme that can digest epithelial cell surface components. This, in turn, allows physical migration into/between epithelial host cells. The second mechanism is the epithelial cell endocytosis of *C. albicans*. During this process, *Candida* can damage the host epithelium and the host’s immune system [19,66,67]. Furthermore, *Candida* can produce carcinogenic compounds, nitrosamines, N-nitrosobenzylmethylamine. Strains with high nitrosamines were isolated from lesions with advanced precancerous changes. In such cases, the yeast cells expand from the mucosal surface toward epithelial cells that exhibit the ability to transport and deposit precursors, nitrosamines, into the deep layer, leading to epithelial dysplasia [65]. An in vivo study reported the carcinogenic susceptibility of salivary glands to nitrosamine [68]. It has also been reported that the workers exposed to nitrosamine have a higher mortality rate from salivary gland carcinoma [69]. 

## 4. *Candida* Infection and Salivary Gland Function

### 4.1. Candida Infection Affecting Salivation

In the early stages of oral candidiasis, *Candida* attaches to the oral mucosa and begins to multiply [64,70]. Several proteins, such as secretory IgA, lactoferrin, histatins, and defensins, downregulate adhesion and multiplication of *Candida* [10,71,72,73]. Among the proteins, histatins and defensins are particularly effective as antifungal factors, which are produced in epithelial cells and salivary glands [74]. Histatins 1, 3, and 5 are present within saliva, accounting for about 80% of total salivary histatins [75]. Human β-defensin-1 was isolated from both the major and minor salivary glands, especially from ductal cells and not acinar cells [76]. Therefore, when salivation or the antifungal agent levels of saliva are reduced, oral microbial hyperproliferation is permitted and oral candidiasis can more easily manifest [73].

Salivary flow showed a significantly negative correlation between stimulated salivary flow rates and *Candida* colony-forming units (CFUs) in the patients with xerostomia [77]. This trend also appeared in patients with reduced salivation after radiation therapy [78]. Antifungal therapy for candidiasis patients can expect to relieve pain, redness, and oral mucosa atrophy. Notably, antifungal therapy often increases the amount of saliva by removing *Candida*. A clinical study investigated the effects of *Candida* elimination on stimulating whole salivary flow rate [79]. The patients with successful elimination of *Candida* showed significantly increased stimulated whole salivary flow rate, whereas patients with unsuccessful elimination of *Candida* did not show increased stimulated whole salivary flow rate. Sympathetic stimuli, like acute pain and stress from *Candida* infection, can reduce salivary flow rate. In other words, parasympathetic stimuli result in increased saliva flow rate; on the other hand, sympathetic stimuli result in more viscous saliva secretions [24,80]. On the basis of this evidence, researchers have suggested that the increased stimulated whole salivary flow rate after treatment was the result of reduced sympathetic stimulation by oral pain reduction [79]. The study states that a decrease in sympathetic stimulation could lead to changed watery salivary secretion. However, 13.5% of the patients with successful elimination of *Candida* did not show increased stimulated whole salivary flow rate [79]. The unrestored salivary flow rate may be a result of salivary gland destruction from the *Candida* infection, and the salivary glands could not restore function even after successful *Candida* treatment [20,62]. 

### 4.2. Candida Infection and Host Immune Response

Oral *Candida* infection on salivary glands causes host immune responses by activation of T lymphocytes. The T cells mediate inflammation by stimulating the production of inflammatory cytokines, such as TNF-α, IL-1ß, and IL-6. These T cells also stimulate the production of inflammatory chemokines and recruit neutrophils and macrophages. The rapid and localized induction of these cytokines form the first line of defense that limits the transmission of invading *Candida*. However, recurrent or chronic infections can provide an elevated inflammatory environment, leading to organ dysfunction [81]. TNF-α and IL-1ß play well-known roles in the pathogenesis of chronic inflammatory diseases. These cytokines may affect salivary gland damage [82,83,84]. The role of these cytokines in the etiology has been determined experimentally in Sjögren’s syndrome with dry mouth [85]. TNF-α suppresses the transcription of Aquaporin-5 and destroys human salivary gland acinar cells. Aquaporin-5 is critical for saliva production and a specific channel protein found in the acinar cells that allows for rapid transcellular migration of water in response to an hydrostatic/osmotic pressure gradient [86,87]. There is a cycle of destruction where *Candida* causes immune mediated salivary gland destruction, following reduced salivary flow and consequent *Candida* infection.

## 5. Diagnosis of Oral Candidiasis

### 5.1. Tentative Diagnosis Using Clinical Features and Characteristics

**Figure 3:**
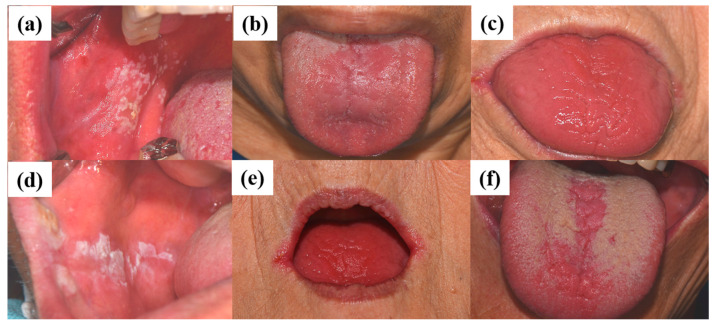
Clinical manifestations of oral candidiasis: (**a**) acute pseudomembranous candidiasis, (**b**) acute atrophic candidiasis, (**c**) chronic atrophic candidiasis, (**d**) chronic hyperplastic candidiasis, (**e**) angular cheilitis, and (**f**) median rhomboid glossitis. Clinical photographs were taken under patients’ informed-consent agreement, with approved Institutional Review Board, PNUDH-2017-026, from the Pusan National University Dental Hospital.

The diagnosis of oral candidiasis can usually be made through a complete medical history and physical examination [88]. Most commonly, candidiasis demonstrates as acute pseudomembranous candidiasis, acute atrophic candidiasis, chronic atrophic candidiasis, chronic hyperplastic candidiasis, angular cheilitis, or median rhomboid glossitis (Figure 3) [89]. (a) Pseudomembranous candidiasis accounts for approximately 35% of oral candidiasis cases. In these cases, the pseudomembrane can be easily removed, revealing underlying mucosa, with minimal bleeding. The pseudomembranous white matter consists of debris, fibrin, and exfoliated epithelium invaded by *Candida* and its hyphae. Acute pseudomembranous candidiasis can be chronic, either intermittently or constantly affecting the patient. The condition may occur in infants, immune-compromised patients (leukemia and AIDS), or patients taking medication such as antibiotics, immunosuppressants, or topical corticosteroids [18,90,91]. (b) Acute atrophic candidiasis, also known as erythema candidiasis, is usually associated with a burning sensation in oral mucosa. It presents as a raw-looking red lesion and occurs prior to the formation of the pseudomembrane or appears after the removal of the pseudomembrane. Acute atrophic candidiasis usually occurs on the dorsal surface of tongue and is characterized by absent papilla due to the use of topical antibiotics or systemic long-term corticosteroids or antibiotics [18,90,91]. (c) Chronic atrophic candidiasis, referred to AS “denture stomatitis”, is usually associated with wearing dentures and inhibited salivary flow. It appears as erythematous inflammation and edema in denture occluded areas. These lesions are caused by dentures rubbing against the oral mucosa, creating a moist and warm environment that is ideal for the growth of *Candida*. Chronic atrophic candidiasis can be symptomatic, causing soreness and burning, or asymptomatic and only found on routine examination [92,93]. (d) Chronic hyperplastic candidiasis is a rare type of oral candidiasis and appears as a rough or nodular lesion, which complicates the diagnosis by differentiating from oral cancer. It typically appears as white patches on the commissures of the oral mucosa. The main cause of chronic hyperplastic candidiasis is *C. albicans*, but other systemic co-factors, such as vitamin deficiency and generalized immune suppression, may contribute. Clinically, the lesions are asymptomatic and regress after proper antifungal treatment and correction of underlying nutritional deficiencies or other co-factors. If the lesion is not treated, it can develop into dysplasia or carcinoma [94]. (e) *Candida*-associated angular cheilitis is inflammatory fissures that emanate from the commissure of the mouth. Angular cheilitis is frequently found in the clinic, including cases involving a combination of *Candida* and bacterial organisms. Signs and symptoms may include bleeding, blisters, cracks, crusts, itchiness, pain, redness, and swelling. Predisposing factors can be loss of vertical height in the denture wearer, habitual lip licking, mouth breathing, or nutritional deficiencies, particularly with vitamin B12 or iron [95]. (f) Median rhomboid glossitis is a term used to describe the area of a smooth, red, flat, or raised nodule in the middle of the dorsal surface of the tongue. The affected area of the tongue usually does not have a normal coating of filiform papilla covering the entire upper surface of the tongue. High levels of *Candida* can be discovered from these lesions, which are associated with the frequent use of steroid inhalers or cigarettes [18,90,91].

### 5.2. Definite Diagnosis Using Cytology and Culture

Diagnosis can be confirmed by smear, oral rinse sample, whole saliva sample, culture, or oral biopsy [88]. Specimens for cytology can be obtained by scraping the lesion with a tongue blade. PAS staining of specimens reveals the existence of *Candida* hyphae and budding yeast. Moreover, 10% potassium hydroxide (KOH), gram, and methylene blue staining can be used instead of PAS. The sensitivity of smear is 51.6%, which is less than that of sample (oral rinse or whole saliva sample) culture. *Candida* species at a low concentration of 200 to 500 cells per milliliter of saliva could be detected using cell culture method rather than cytology method. Of the asymptomatic healthy population carry *Candida* in the oral cavity. Therefore, it is necessary to identify a threshold amount of *Candida* species (>270 CFU/mL), to distinguish oral candidiasis from oral carriage [96]. A definitive diagnosis of candidiasis requires the confirmation of tissue invasion by *Candida*, using biopsy with PAS staining. Biopsies are always required in hyperplastic candidiasis in order to discard the existence of epithelial dysplasia [97].

## 6. Prevention and Treatments of Oral Candidiasis

### 6.1. Prevention

Clinicians should notice that patients with immunocompromised disease, such as AIDS and diabetes, or individuals who have the risk factors of usage of medication (antibiotics, steroids, or immunosuppressants), impaired salivary gland function, dentures, poor oral hygiene, or a high-carbohydrate diet can develop candidiasis easily. Therefore, periodical oral examinations, oral hygiene instruction, and periodic prophylaxis could prevent oral candidiasis. Oral hygiene includes cleaning the tongue with a tongue cleaner, cleaning teeth and dentures with a toothbrush [98], and rinsing oral mucosa with chlorhexidine. In addition, dentures should be removed at night and meticulously washed and soaked in a disinfectant solution, such as chlorhexidine and sodium hypochlorite (1%) [15,99]. To reduce the destruction of salivary glands due to repetitive candidiasis, periodical oral examinations with prophylaxis and proper oral hygiene instruction should be recommended and practiced. 

### 6.2. Treatments of Candida Infection

The treatment of oral candidiasis is based on four basic principles [98,100]: Assess the *Candida* infection type, diagnose the infection early and accurately, correct the predisposing factor or underlying disease, and administer antifungal agents appropriately. In order to select the proper medications, studies consider factors including local or systematic approach, type of *Candida,* clinical findings [90], and medication efficacy and toxicity [101]. Commonly used antifungal medications are included in Table 1. 

Based on the histopathological information via microscopic examination and fungal culture, clinicians should choose the most appropriate antifungal medication. Polyene was the first broad spectrum antifungal agent discovered in the 1940s and 1950s [102]. Polyenes, such as nystatin and amphotericin B, bind to and weaken ergosterols in fungal cell membranes that can initiate the leakage of K+ and Na+ ions, thus contributing to fungal cell death. Polyenes are considered fungicidal and have broad activity against most fungal organisms. Amphotericin B is an antifungal drug used for serious fungal infections and nystatin is used to treat *Candida* infections of the skin, vagina, mouth, and esophagus [102,103]. Although resistance to polyene medication is rare, some fungal species exhibit intrinsic resistance to polyenes [104,105]. Nystatin is only effective topically, and amphotericin B, which is effective orally and intravenously, is well-known for its severe and potentially lethal side effects such as high fever, kidney damage, and multiple-organ damage. The search for antifungal agents with an acceptable toxicity profile first led to the discovery of azole. Therefore the first azoles were discovered in 1944, but were not approved for use in humans until the early 1960s [102]. Azoles inhibit 14-α-sterol demethylase, a cytochrome P-450 enzyme involved in ergosterol synthesis [106], resulting in the accumulation of toxic sterol intermediaries and loss of membrane integrity. Most azoles are fungistatic and have a broad spectrum against filamentous fungi and yeasts [107,108]. The search for azole antifungal agents with an acceptable toxicity profile led to the discovery of the first ketoconazole. Later, the triazoles fluconazole and itraconazole were developed with an improved safety profile and comparatively broader range of antifungal activity. Analogs have been developed to overcome limitations, such as a suboptimal spectra of activity, need to develop resistance, unfavorable pharmacokinetics, drug–drug interactions, and toxicity [109]. *Candida* species resistance to the azole medications (e.g., itraconazole, clotrimazole, and fluconazole), including *Candida glabrata, Candida tropicalis,* or *Candida parapsilosis* are susceptible to polyene medication. Polyene medications are not well absorbed from the gastrointestinal tract but are effective for topical application [89]. Topical antifungal therapy is recommended as the primary treatment option for mild cases of *Candida* infection. If the lesion is refractory to topical treatment or recurs frequently, systemic antifungal therapy is suggested. However, systemic antifungal therapy must be considered as the primary treatment for patients with immunocompromised conditions due to the risk of candidemia [103].

The removal of *Candida* biofilm is necessary, in combination with appropriate medication. Successful treatment of candidiasis depends upon biofilm control, using daily oral hygiene and professional prophylaxis. The *Candida* biofilm is a thick extracellular polymeric substances layer with a dense network of yeasts, pseudohyphae, and hyphae [110]. The biofilm allows *Candida* to easily attach between cells and other surfaces, such as dentures. The biofilm provides barriers between *Candida* and the surrounding environment, thus protecting *Candida* from antifungal medications [90]. Therefore, the removal of *Candida* biofilm from the dentures, as well as from all sides of the oral cavity, contributes to lowering the failure rate of antifungal treatment; it is essential for the effective treatment of *Candida* infection. 

### 6.3. Treatments of Salivary Gland Dysfunction

#### 6.3.1. Symptomatic Management

Hyposalivation is symptomatically managed with methods such as lifestyle changes, synthetic saliva supplementation, salivary gland stimulants, and the use of sialagogues (e.g., muscarinic receptor agonists, pilocarpine, and sevimeline) to elevate the flow rate of saliva [111,112]. Among the sialagogue treatment options, pilocarpine is the most commonly selected medication to increase saliva secretion by stimulating the salivary gland. However, pilocarpine’s effect is temporary and causes side effects, including excessive sweating and tearing, chills, dizziness, flushing, nasal congestion, vocal changes, nervousness, tremors, and diarrhea [113,114]. To improve the side effects of pilocarpine, consistent and controlled release of pilocarpine in the salivary glands was considered [115]. Controlled drug-release systems have been developed and are expected to deliver therapeutic agents directly to salivary glands using novel biomedical approaches, such as hydrogels [116], polymer-based microchips [117], nanoshells [118], and microfluidics technology [119]. For example, polymer hydrogels for controlling pilocarpine release have already been clinically tested in patients with Sjögren’s syndrome [115]. However, the polymer hydrogel and associated medication could not improve the discomfort of patients who had completely destroyed acinar cells. Since the severity of salivary dysfunction may vary from patient to patient [115], the literature suggests that the most effective therapy depends on the evaluation of salivary glands damage. 

#### 6.3.2. Gene Therapy and Cellular Stimulation

Gene delivery therapy could be applied to salivary gland cells to ameliorate salivary gland function. Loss of functional water channels in salivary gland epithelia is often considered one of the hallmarks of salivary gland dysfunction, and recent advances are aimed at restoring the permeability in an attempt to increase saliva production. Gene therapy was attempted to deliver the human aquaporin 1 (AQP1) gene to the salivary gland via recombinant adenovirus delivery (AdhAQP1) in rats [120]. The result suggests that AQP1 gene transfer may have potential as an approach for the treatment of salivary hypofunction. In the human study, five of 11 patients experienced elevated salivary flow 3–4.7 years after the AdhAQP1 vector delivery treatment [121]. Clinically, gene delivery to salivary glands offers the accessibility of transfer vectors in a less invasive manner [122]. The administration of bioactive components, cells, and genes directly into the salivary gland is a promising therapeutic option, when salivary-gland cells are intact. Systemic and local delivery was performed to administer a multitude of reagents, including adenoviral vectors, primary cells, growth factors, antioxidant compounds, and cytokines [123,124,125,126,127,128,129]. In addition, bone-marrow-derived cell (BMC) recruitment by cytokine stimulation has been reported for recovery of salivary gland cells in vivo [130,131]. Studies demonstrate that subcutaneous injection of granulocyte colony stimulating factor mobilized BMC into the bloodstream and induced migration of BMC to the damaged salivary gland, resulting in improvement of morphology and function of the submandibular salivary gland [130]. 

#### 6.3.3. Stem Cell Therapy 

Among the stem cell approaches, a majority of research relies on mesenchymal stem cells (MSCs) [132,133]. In the MSC salivary transplant in vivo studies, MSCs have been acquired from bone marrow, adipose tissue, or umbilical cord blood [132,134,135]. MSCs are able to be harvested in a non-invasive manner, with relative abundance. Although the differentiation of MSCs into salivary gland acinar cells has been observed in vitro, the actual contribution to differentiation in vivo is unclear and controversial. Their beneficial action can occur primarily through paracrine pro-survival/proliferative effects on the remaining local stem/progenitor cells and cells of the surrounding environment [136,137]. However, MSCs have primary safety concerns, including unknown long-term report, tumorigenic, and metastatic potential. In addition, donor-dependent efficacy and heterogeneous properties of MSCs pose critical obstacles [138]. Therefore, autologous stem cells are preferred to repair salivary gland function. Transplantation of pluripotent salivary gland-specific epithelial stem/progenitor cells has been shown to morphologically and functionally repair salivary gland tissues. Multi-level of potency from the salivary gland cells could be applied to repair compartments of the salivary gland, recover the secretory compartment conditions, and maintain the secretory compartment [139,140]. Permanently differentiated and post-mitotic acinar cells may be able to self-duplicate after damage in post-chronic sialadenitis [141], post-duct ligation [142], partial salivary gland excision [143], and post-radiation therapy. In fact, most patients requiring autologous cell therapy are elderly and do not have enough stem/progenitor cells [144,145]. Although the number of stem/progenitor cells was increased by using heparin sulfate–stimulated growth factors [146] or Aldehyde dehydrogenase-3 activator [147], the absolute number of stem/progenitor cells required for functional regeneration of the human gland has not been clearly defined. 

#### 6.3.4. Tissue Engineering

In cases of full destruction of salivary glands, it is insufficient to restore part of the damaged salivary glands and their function. To achieve a complete functional replacement of lost or damaged tissue, tissue-engineered organoids to reconstruct fully functional organs has been proposed [148,149]. In vitro tissue-engineered organoids, using three-dimensional biomaterials loaded with salivary gland cells and/or bioactive cues, can be embedded in extracellular matrix to connect with remaining tissue residues. This approach, called the “organ germ method”, has been evaluated for the regeneration of fully functional salivary glands in mice, which has been induced by mutual epithelial and mesenchymal interactions [149]. The bioengineered salivary gland responded to pilocarpine administration and taste stimulation by producing saliva. To be utilized in clinical practice, an appropriate cell source needs to be clearly identified. Recently, induced pluripotent stem cells or embryonic stem cells have been studied in salivary-gland tissue engineering [150]. 

Although notable progress in the treatment of hypofunctional salivary gland has been attempted over the last decades, no definitive treatment has been confirmed. Limitations of in vivo studies for translation to human trial are present due to the biological differences between human and rodent salivary glands and require further study [140]. In addition, potential differences in the development and/or regeneration strategies between different glands (e.g., parotid, submandibular, and sublingual) should be considered for future translation.

## 7. Conclusions

A mutual vicious cycle presents itself between the salivary gland and *Candida* infection: A decrease in the saliva flow rate creates conditions that encourage *Candida* infections, and then the *Candida* infection damages salivary glands, leading to a further decrease in saliva secretion. This pathological malfunction could result in temporary or permanent destruction of the salivary glands and may cause various intraoral symptoms, including dry mouth, speech and swallowing difficulties, and oral infections. Oral candidiasis should be detected early and treated correspondently, using an antifungal agent like nystatin and fluconazole to prevent the development of chronic salivary gland dysfunction. Correction of the underlying disease, biofilm control by using tongue cleaners, and chlorhexidine rinses must be accompanied. Limitation of current treatment options of salivary gland dysfunction are symptomatic management with medication with their side effects. Therefore, scientists have made an effort to regenerate salivary glands, using various sources to overcome the limitations of current treatments. Regeneration of salivary glands has been attempted by the activation of remaining cells with growth factors, genes, cytokines, and the transplantation of progenitor cells and mesenchymal stem cells. However, these technologies are still limited in clinical application and are in a stage that requires further research. Therefore, the clinician’s role in the early detection and proper treatment of vulnerable populations who could be exposed to *Candida*-mediated salivary gland dysfunction is important.

## Figures and Tables

**Table 1:** Summary of the antifungal medications and their side effects.

Drug	Formulation	Dose	Side Effect
Amphotericin B	Infusion 50 mg	100–200 mg/6 h	Renal, cardiovascular, spinal, and neurological
Nystatin	Suspension 60 mL	4–6 mL/6 h	Well tolerated
	Ointment 30 g	2–4 times/day	
	Tablets	2 every 8 h	Uncommon nausea, vomiting, and gastrointestinal effects
Clotrimazole	Gel 1%	3 times/day	Occasionally skin irritation and burning sensation.
	Tablets 10 mg	5 times/day	
Miconazole	Gel	100 mg/6 h	Uncommon burning, irritation, nausea, and diarrhea.
Ketoconazole	Gel 2%	3 times/day	Nausea, vomiting
	Tablets	200 mg, 1–2/day	abdominal pain.
	Suspension 30 mL		
Fluconazole	Tablets	50–100 mg/day	Nausea, vomiting, diarrhea, and abdominal pain.
	Suspension	10 mg/mL	
Itraconazole	Capsule	100–200 mg/day	Nausea, vomiting, diarrhea, and abdominal pain.

Table was adapted with permission of CEDRO, Centro Espanol de Derechos Reprograficos, from “Current treatment of oral candidiasis: A literature review”, 6, 5, 2014 [98]); permission conveyed through Copyright Clearance Center, Inc. (Danvers, MA, USA).

## Data Availability

Data sharing not applicable.
